# 

**Published:** 1884-09

**Authors:** 


					﻿SEPTEMBER, 1884.
OLD SERIES
Vol. xxv.
4^ 1855 %
JSLEiaCSERIES
7-
Mmhf
IffiDIGJL-SUfflQ L
40URNAM
Wtbu 4 1//C. dt).
■y^bv1
W. F. WESTMOREL AND, MwS
H.V.M.MILLER,M.D.LL.D.Jk
JAMES A.GRAY,
PUBLISHED BY JAMES P.HARRISON&CO
HP	Atlanta,ga.
SUBSCRIPTION: 2.50 A YEAR.
				

